# Supplementary material for: Identification and functional analysis of non-coding regulatory small RNA FenSr3 in Bacillus amyloliquefaciens LPB-18
Source: PeerJ. 2023 May 15;11:e15236. doi: 10.7717/peerj.15236 (PMC10194069; doi:10.7717/peerj.15236)
Supplement: Supplemental Information 4 [file peerj-11-15236-s004.zip › KO/CK-vs-T1_map/map04014.html]

KEGG PATHWAY: Ras signaling pathway - Reference pathway


|  |  |
| --- | --- |
| **Ras signaling pathway - Reference pathway** |  |

[
Pathway menu
| Organism menu
| Pathway entry
| Show description
| User data mapping
]

The Ras proteins are GTPases that function as molecular switches for signaling pathways regulating cell proliferation, survival, growth, migration, differentiation or cytoskeletal dynamism. Ras proteins transduce signals from extracellular growth factors by cycling between inactive GDP-bound and active GTP-bound states. The exchange of GTP for GDP on RAS is regulated by guanine nucleotide exchange factors (GEFs) and GTPase-activating proteins (GAPs). Activated RAS (RAS-GTP) regulates multiple cellular functions through effectors including Raf, phosphatidylinositol 3-kinase (PI3K) and Ral guanine nucleotide-dissociation stimulator (RALGDS).

|  |  |  |
| --- | --- | --- |
| Reference pathway | 184% 150% 122% 100% 82% 67% 55% | 图片下载 |
